# Prevalence and Temporal Characteristics of Housing Needs in an Urban Emergency Department

**DOI:** 10.5811/westjem.2020.9.47840

**Published:** 2020-12-07

**Authors:** Leah Fraimow-Wong, Jennifer Sun, Partow Imani, Daniel Haro, Harrison J. Alter

**Affiliations:** * University of California San Francisco, School of Medicine, San Francisco, California; † Highland Hospital – Alameda Health System, Department of Emergency Medicine, Oakland, California; ‡ University of California Berkeley, School of Public Health, Berkeley, California; § New York University, Graduate School of Arts and Sciences, New York, New York; ¶ Andrew Levitt Center for Social Emergency Medicine, Berkeley, California

## Abstract

**Introduction:**

Our objective was to determine the proportion of patients in our emergency department (ED) who are unhoused or marginally housed and when they typically present to the ED.

**Methods:**

We surveyed patients in an urban, safety-net ED from June–August 2018, using a sampling strategy that met them at all times of day, every day of the week. Patients used two social needs screening tools with additional questions on housing during sampling shifts representing two full weeks. Housing status was determined using items validated for housing stability, including PRAPARE, the Accountable Health Communities Survey, and items from the United States Department of Health and Human Services. Propensity scores estimated differences among respondents and non-respondents.

**Results:**

Of those surveyed, 35% (95% confidence interval [CI], 31–38) identified as homeless and 28% (95% CI, 25–31) as unstably housed. Respondents and non-respondents were similar by propensity score. The average cumulative number of homeless and unstably housed patients arriving per daily 8-hour window peaks at 7 AM, with 46% (95% CI, 29–64) of the daily aggregate of those reporting homelessness and 44% (95% CI, 24–64) with unstable housing presenting over the next eight hours.

**Conclusion:**

The ED represents a low-barrier contact point for reaching individuals experiencing housing challenges, who may interact rarely with other institutions. The current prevalence of homelessness and housing instability among urban ED patients may be substantially higher than reported in historical and national-level statistics. Housing services offered within normal business hours would reach a meaningful number of those who are unhoused or marginally housed

## INTRODUCTION

### Background

Homelessness, housing, and health are deeply intertwined. Data suggest the annualized risk of death in homeless individuals to be 7.2%, 1.6 times that of housed individuals after adjustment.^1^ The experience of homelessness is also related to significantly elevated rates of chronic disease, disability, and infection, making homelessness an issue of profound concern in public health and for health systems.^2^–^4^ From a health systems standpoint, individuals experiencing homelessness have higher rates of emergency department (ED) utilization compared to those who are housed.^5^–^7^ Previous data suggest that ED utilization rates correlate with changes in housing status, suggesting that a history of homelessness may increase the risk for higher ED use.^5^,^8^ National data on ED visits by homeless individuals show that they are more likely to have arrived by ambulance, to lack insurance, and to have had a recent ED visit or hospitalization.^9^,^10^

### Significance

A natural, if somewhat revolutionary, response to the health risks of homelessness has been a national movement toward “housing as healthcare.” As described in a 2013 paper in the *New England Journal of Medicine*, this movement embraces the deployment of resources typically reserved only for the provision of medical care toward housing, where these resources could potentially have a more substantial and lasting impact on health.^11^ Because of the role that the ED plays in providing medical care to people experiencing homelessness, the ED may represent a natural site to practice a housing-as-healthcare model. In addition, because the ED is so frequent a source of care for chronically ill individuals experiencing homelessness, those who have started on the road to permanent supportive housing but are lost to the system could be reconnected during their ED visit.

Despite this area of opportunity, little is known about the true prevalence of homelessness and housing instability in urban EDs, in particular in safety-net EDs, which the Institute of Medicine defines as those that “care for a proportionately greater share of poor and uninsured people.”^12^ There is also little research on the temporal pattern of ED usage by individuals experiencing homelessness or housing instability. A recent systematic review on homelessness in the ED found that it is likely under-recognized, and prescribed more research on its prevalence and characteristics.^13^

Such a view acknowledges that historical and national-level data on homelessness may be poor indicators of current urban ED housing needs, especially with many major metropolitan areas experiencing significant housing crises in recent years. Furthermore, few studies have looked at the prevalence of housing challenges overall within the ED, examining both homelessness and unstable housing. Housing instability may present a significant area of opportunity for homelessness prevention linkage services in EDs. Finally, temporal findings could have important practical implications for co-locating housing linkage services within the ED, as has been done for human immunodeficiency virus, hepatitis C, and medication for opioid use disorder.^14^–^16^ The workflow of the typical ED is poorly synchronized with most employment norms, and how to staff such a position in a way to best capture the need has been a conundrum within the field of social emergency medicine.

### Study Aim

Our study was dually aimed to determine the proportion of visitors to a safety-net ED who are unhoused or marginally housed and to determine whether a greater number of such patients present to the ED at certain times of the day or the week. As housing needs are only one area of health-related social needs, we also gathered information on other social needs of our study population.

**Population Health Research Capsule**

What do we already know about this issue?

*Emergency departments (ED) serve many patients experiencing homelessness or unstable housing. These needs can profoundly affect health and disease*.

What was the research question?

To best target services, we asked what proportion of ED patients have housing needs and when are they visiting the ED?

What was the major finding of the study?

*Nearly 2 of 3 patients in our ED were homeless or unstably housed. Almost half arrived between 7 AM–3 PM*.

How does this improve population health?

*Unstable housing is a public health crisis broadly affecting ED patients. EDs could be accessible linkage points offering housing services during daytime hours*.

## METHODS

### Study Design

We assessed housing status and social needs of our patients at Highland Hospital ED, an urban safety-net ED with 68,000 annual visits, through a combination of surveys and chart review. To evaluate as representative a sample of the ED population as possible, we sampled at all times of day, every day of the week, covering a period equivalent to two full weeks for a total of 336 hours (14 days ^*^ 24 hours/day) from June 2018–August 2018. All patients at Highland ED during study hours were considered for eligibility. Patients completing the survey section were 18 years of age or older and spoke English or Spanish. Patients who were medically unstable, unresponsive, or had altered mental status were not surveyed, nor were those who had already participated. The work was reviewed and approved by the institutional review board at Highland Hospital.

The survey instrument included two social needs screening tools: 1) PRAPARE: Protocol for Responding to and Assessing Patient Assets, Risks, and Experiences, developed by the National Association of Community Health Centers, Inc., and its partners^17^; and 2) the Accountable Health Communities (AHC) Health-Related Social Needs Screening Tool, developed by the Centers for Medicare & Medicaid Services.^18^ In addition, we developed an item set of questions specifically focused on housing to better understand our patients’ housing situations. These questions are largely sourced from the US Department of Health and Human Services (DHHS).^19^

Trained research assistants (RAs) approached patients during gaps in care and obtained verbal consent using a standard approach script. RAs approached available patients in order of arrival time, circling back to patients who were unavailable at the time of their initial approach when possible. Survey responses from participants were input directly into a secure electronic data capture system, REDCap, on a password-protected tablet.^20^,^21^ RAs read the questions aloud, or participants completed the survey directly on the tablet if preferred. Arrival and discharge times, disposition, medical history, prior ED utilization, and past admissions were abstracted from the electronic health record (EHR) (Wellsoft Corporation, Somerset, NJ)) during a standardized chart review. An aggregate measure of housing status was determined using items validated for housing stability, including PRAPARE, the AHC screening tool, and items from DHHS. We categorized subjects as homeless or unstably housed based on the criteria specified in Table 1. Any patients who did not meet the criteria for the categories of homeless or unstably housed, we categorized as stably housed.

To identify whether there were any substantive differences between patients who completed the survey and potentially eligible patients who did not, we performed an analysis of non-respondents using data from the EHR. We defined non-respondents as those who were approached but declined to respond as well as potentially eligible patients who were not approached. We excluded patients who were found to be ineligible when approached and those with medical records clearly indicating that they did not speak English or Spanish. Respondents were compared 1:1 to randomly selected non-respondent ED patients with visits during the study time period, matched by hour of arrival. We created a propensity score using the following covariates between the two groups: age; gender; acuity; language; race; insurance type (including “other public,” which includes county-pay, workers’ compensation, and others); disposition; past medical history; whether the patient was in custody or on a psychiatric hold; whether there were any indicators of homelessness documented in the clinicians’ chart; past 12-month ED usage; and past 12-month inpatient hospitalizations.

### Statistical Analysis

For each of the three groups, homeless, unstably housed, and stably housed, we calculated standard descriptive statistics such as mean, standard deviation, range, and proportions. We then tested for differences between the groups for each variable using analysis of variance for continuous variables and chi-square tests for categorical variables. Patient arrivals for a given time of day were tabulated and compared for unstably housed and homeless patients. To address any temporal non-randomization in our sample, results were scaled and weighted based on average arrival times of the ED population as a whole as well as the proportion of the available patients that we were able to approach. We calculated the cumulative number of patients who presented within a moving eight-hour window, stratified by homeless and unstably housed as well as cumulative, and then calculated confidence intervals assuming a binomial distribution with the observed probability of an individual’s arrival time falling within the given eight-hour window. To compare our respondent to our non-respondent populations, we calculated propensity scores for each individual based on covariates available for both sets.

## RESULTS

**Figure 1:**
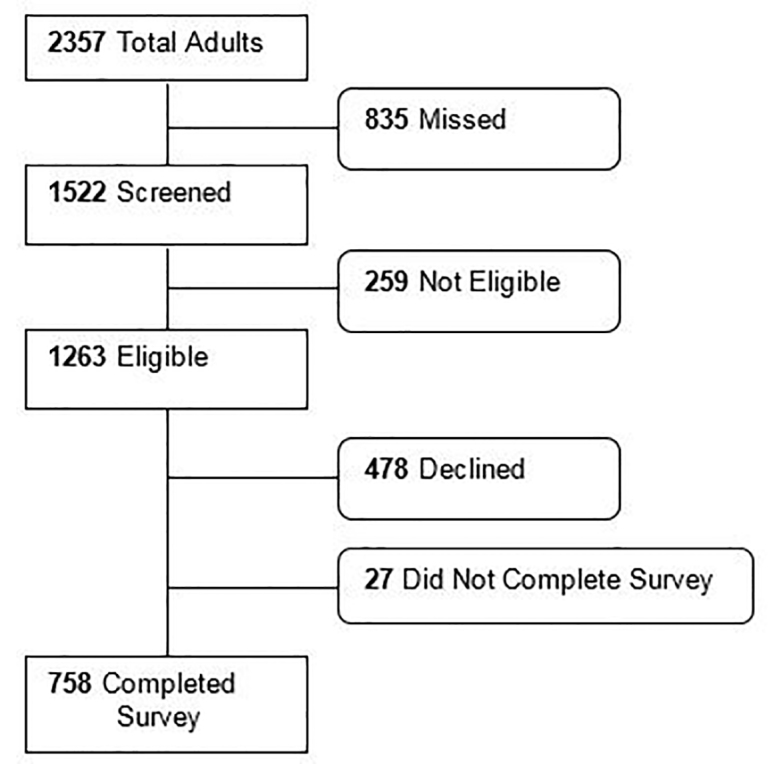
Study flow showing adult patients approached for survey participation.

During the survey times, 2573 ED visits by 2357 unique adults occurred. Among these, we approached 1522. Of those approached, 758 patients completed the survey, 27 started but did not complete the survey, 478 declined to participate, and 259 were discovered to be ineligible after approach (Figure 1). The primary reasons for ineligibility were that the patient 1) could not complete the survey in English or Spanish (51%), or 2) lacked capacity due to altered mental status or critical illness (47%).

Among respondents, 40% identified as Latinx, 39% Black, 15% White, 5% Asian, and 8% other races/ethnicities. Median age was 42 (IQR 29–57) and 54% were male. By our aggregate measure, 35% (95% CI, 31–38) were found to be homeless and 28% (95% CI, 25–31) reported being unstably housed (Table 2).

The rates of homelessness indicated by each survey individually were 26% (PRAPARE), 24% (AHC), and 29% (modified DHHS). (Table 3)

Participants reporting homelessness were more likely to be Black and to report a physical or mental disability. Participants reporting being unstably housed were more likely to be Latinx and to speak a primary language other than English. Stable housing was associated with more than a high school education and with advanced age. All groups reported high levels of Medicaid and uninsurance, typical of our hospital and others in the safety net.

**Figure 2:**
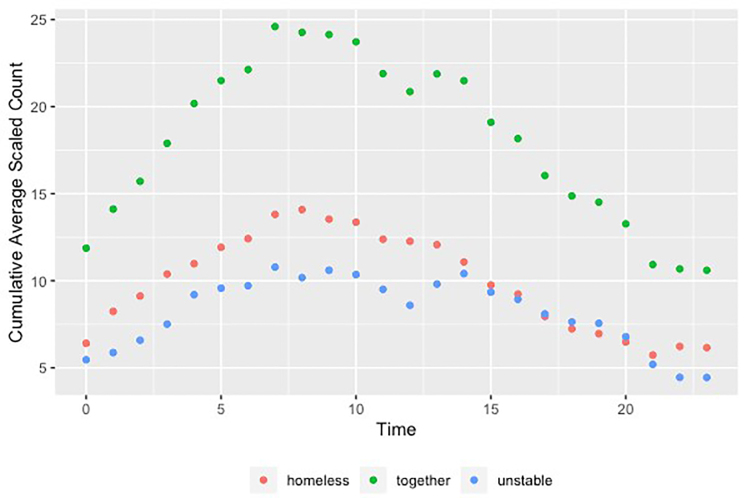
Cumulative average scaled count of homeless and unstably housed in emergency department arrivals 8-hour window. Each hour marking on the x-axis represents the start time of the 8-hour time windows, with the corresponding y value showing the cumulative number of patients who arrived during that 8-hour window by group. 0 = midnight, otherwise numbers denote military time hours.

The adjusted average number of patients arriving per hour who reported being homeless or unstably housed clustered in the hours between 7 am–8 pm (not shown). The average cumulative number of homeless and unstably housed patients arriving per each eight-hour window of the day peaked at 7 am, with 46% (95% CI, 29–64) of the daily aggregate of those reporting homelessness and 44% (95% CI, 24–64) with unstable housing presenting over the next eight hours (Figure 2).

**Figure 3:**
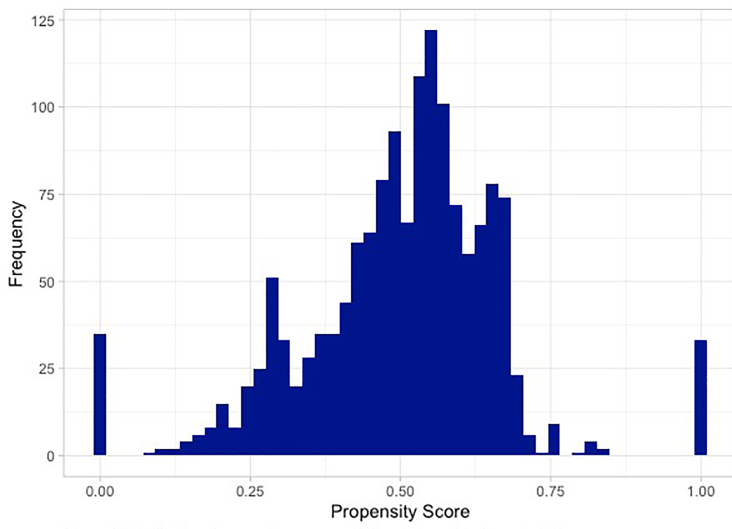
Distribution of propensity scores, which correspond to the probability of an individual having received and completed the survey given their particular combination of covariates.

To investigate whether our respondents were similar to non-respondents, we calculated propensity scores, which indicate the probability that a given patient responded to the survey given their covariates. The distribution of the scores with the mass appearing toward the middle suggests that respondents and non-respondents were relatively similar with respect to baseline covariates (Figure 3). Given the high-dimensional nature of the covariates, it is not surprising to see blips towards the tails.

## DISCUSSION

### Prevalence of Homelessness

In our study, 35% of respondents indicated that they were experiencing homelessness and 28% indicated that they were experiencing housing instability, a substantially higher prevalence than was reported in most previous studies.^5^,^22^–^24^ Indeed, taken together, patients with housing challenges represented the majority of all visits to this urban safety-net ED. Although studies of data from the National Hospital Ambulatory Medical Care Survey (NHAMCS) consistently show rates of homelessness under 1% of all visits,^13^ focused, survey-driven results from more recent studies indicate the prevalence of homelessness to be significantly higher, with wide variability across hospital types. A study of a public ED in New York City using data from 2016–2017 found 21.4% of patients screening positively for homelessness.^24^ Similarly, a survey-based study in Pennsylvania from 2015–2016 reported homelessness rates varying from 7.5% of all visits at a suburban ED to 18.8% at an urban ED.^5^,^22^ A similar order of magnitude was uncovered by Doran et al, who found that 14% of patients in their urban ED were living in shelters or on the streets.^23^

While it is unsurprising that our homelessness rates were higher than NHAMCS data, it is remarkable that they were considerably higher than rates reported in other studies. One possible explanation for the high prevalence we found is that we asked participants about their housing situation in more than one way and classified them as homeless if they met any of the homeless screening criteria from our three surveys. We chose this broader definition of homelessness in an attempt to capture all patients likely to be experiencing homelessness, under the logic that even a patient on the brink of homelessness could benefit from housing services in the ED. Yet even using only the narrower definitions of the established surveys (PRAPARE, AHC) individually, our rates were somewhat higher than those previously reported, with PRAPARE at 26% and AHC at 24%.

In addition to our broader definition of homelessness, our higher rates may be partially explained by our setting: an urban safety-net ED in the San Francisco Bay Area. The Bay Area region has the third highest number nationally of people experiencing homelessness, after New York and Los Angeles.^25^ Our region in 2019 was experiencing a severe housing crisis. A comparison of American Community Survey (ACS) data from 2009 and 2017 shows the average median rental price increasing by 33% in Alameda County where our survey was conducted.^26^ As of 2017, the most recent published year of results for the ACS, 86% of the county’s renter households earning less than $50,000 spend over 30% of their household income on housing.^26^ In addition, urban public EDs likely serve a higher proportion of homeless and lower income patients than private or rural hospitals, factors that might contribute to higher reported rates of housing instability and homelessness than EDs on average nationwide. Nonetheless, given that more than half of those experiencing homelessness live in cities, our results may be relevant to the institutions and communities where those experiencing homelessness and housing instability are most likely to receive their emergency care.^26^ As of publication, our study protocol is being expanded to other hospitals in other regions of the US.

### Housing Instability

While many studies have focused on patients in the ED who are experiencing homelessness, far fewer have studied the prevalence of housing instability among ED patients. Individuals experiencing housing instability have been shown to have higher rates of ED and acute care utilization than stably housed individuals.^27^,^28^ Unaffordable housing has also been associated with increased odds of hypertension and cost-related healthcare non-adherence, as well as worse self-rated health compared to controls using propensity score analyses.^29^ In our study, 28% of ED patients reported having unstable housing, suggesting that the ED may offer significant opportunities for homelessness prevention through linkage to legal and social services. The ED as a touchpoint may be of particular import to patients who have limited interaction with other public institutions.

Of note, our results indicate that those reporting housing instability, as opposed to homelessness or stable housing, were significantly more likely to speak a primary language other than English and to report lower rates of English proficiency. Families with limited English proficiency may be particularly vulnerable to predatory or discriminatory housing practices and face higher risks and greater challenges advocating for their rights as tenants.^30^ Given these findings, a more inclusive study on the interplay between housing stability, language access, and the particular challenges faced by low-income immigrant communities may be warranted. Additionally, these findings may be relevant to designing housing assistance programs within the ED that meet the language access needs of potential participants.

### Temporal Patterns of Homelessness and Housing Instability in the ED

Our findings also suggest that the majority of both homeless and unstably housed patients present during daytime hours, with almost half arriving between 7 am – 3 pm. Our temporal trend for homeless and unstably housed patients aligns with previous studies of the general adult ED population showing patient flow to be highest during daytime hours.^31^ One implication of our temporal finding is that its congruence with employment norms could help facilitate the provision of housing services by community agencies in the ED. The 7 am – 3 pm window captured nearly half of all homeless and unstably housed patient arrivals and represented the highest incidence eight-hour interval, followed closely by the slightly more conventional 8 am to 4 pm period. The practical implications may be of particular interest to hospitals in light of California’s newly enacted Senate Bill-1152, a so-called “safe discharge” law, which mandates a coordinated discharge plan for homeless patients including referrals to community agencies.^32^

### Public Health Implications

Taking a step back, our finding that a majority of visits to this urban safety-net ED were made by patients experiencing housing challenges supports the view that lack of affordable, stable housing has become a public health crisis. The deeply intertwined relationship between housing instability, healthcare utilization, and poor health is both intuitive and widely documented in the literature.^33^–^35^ It is our view that if the majority of patients coming to an ED had a particular diagnosis, say kidney disease, vast efforts would be mobilized to better understand and treat that condition. We believe that such efforts are needed in addressing homelessness and housing insecurity.

Our data support the view that the ED presents a unique linkage point in building systems that redefine housing as healthcare. A housing specialist centered in the ED, like other consulting specialists, would diagnose the acuity of a housing emergency—is the health threat of this person’s housing status measured in hours, days, or months?—and leverage resources appropriately. Due to its accessibility, the ED represents a low-barrier contact point for high-cost and hard-to-reach users. The ED may provide particular value in reaching individuals experiencing housing instability and homelessness who interact less frequently with other government systems, whether as a result of historical marginalization, distrust of government actors, language barriers, or other obstacles.^36^–^38^ A longitudinal study of newly homeless persons found that over a third had made a visit to the ED in the year prior to becoming homeless.^39^ Given the high prevalence of housing instability reported by ED patients in our study, the prevention of homelessness through access to legal advocacy, eviction prevention, rent assistance, and other proactive supports arising from the ED may be a significant area of opportunity.

## LIMITATIONS

There are several limitations to this study. First, the study was conducted solely during summer months, which intuitively may impact rates of ED utilization by those facing housing challenges, even though recent studies suggest that seasonal variation may be limited.^32^,^40^ Second, we missed a considerable proportion of our sampling target. Due to resource constraints, only 65% of the patients presenting in our sampling time period were approached for inclusion in the study, and 31% of the patients who were approached then declined to take the survey. Patients were approached in order of their arrival time, but some number of eligible patients did not complete the survey because they were receiving care at the time of the initial approach or were missed due to personnel limitations. Therefore, our study best represents a convenience sampling and a non-consecutive sampling of ED patients. Consecutive sampling would have increased the significance of the study.

Given this issue, a primary concern is that it is possible that a higher proportion of homeless patients were approached or chose to participate in the survey than the general ED population. We attempted to address this limitation by conducting a propensity score analysis comparing the surveyed group with potentially eligible non-respondents who were either never approached or who declined to complete the survey. Our analysis suggests that these groups would have responded similarly. However, this does not replace the value of having a larger and/or consecutive sample, and subject selection remains an important limitation.

An additional limitation involves the patients who were ineligible to participate. Our study did not include patients who could not complete the survey in English or Spanish and patients who could not complete the survey due to critical illness. Out of the 259 patients deemed ineligible after being approached (17% of screened patients), over half were ineligible because they spoke languages other than English or Spanish, and nearly half were ineligible because of critical illness. Finally, before more widespread confirmation, our results should be generalized with some caution, as housing challenges and associated service provision can vary widely geographically.

## CONCLUSION

We found that nearly two-thirds of patients seeking care in our ED faced housing challenges, with 35% homeless and 28% unstably housed. We also found that almost half such patients arrive between 7 am and 3 pm, when they would be accessible to a housing specialist for counseling, referral, and management. All hands on deck are needed to address this crisis, and given the immense health impacts of housing challenges and the substantial financial resources of the healthcare sector, it is becoming increasingly compelling for health systems to be involved. Emergency departments may offer a unique linkage point in filling the “housing as healthcare” prescription.

## Figures and Tables

**Table 1:** Study flow showing adult patients approached for survey participation on homelessness and housing instability in Oakland, California.

Homeless	Unstably housed
“Yes” to any of the following:	Does not meet the criteria for Homeless and “Yes” to any of the following:
“I do not have housing” (PRAPARE) “I do not have a steady place to live” (AHC) “Last night, I stayed at... An emergency shelter, hotel, or motel (whether or not paid for with a voucher)” Transitional housing for homeless persons” A place not meant for human habitation” A friend or family member’s room or apartment” (DHHS) “I am currently homeless” in response to ability to stay in last night’s place for more than 90 days (DHHS)	Yes, I am worried about losing my housing” (PRAPARE) I have a place to live today, but I am worried about losing it in the future” (AHC) Moved 3 or more times in the past 12 months Has had to move in with other people in the past 12 months because of financial problems Unable to stay in current place for more than 90 days (DHHS)

*PRAPARE*, Protocol for Responding to and Assessing Patients’ Assets, Risks, and Experiences; *AHC*, Accountable Health Communities; *DHHS*, US Department of Health and Human Services.

**Table 2:** Baseline characteristics of all respondents by housing status.

	Housed N = 281 (37.1%)		Unstably housed N = 213 (28.1%)		Homeless N = 264 (34.8%)	
			
Sociodemographic characteristics	n	%	n	%	n	%
Age group						
18 – 24 years	44	15.7	20	9.4	36	13.6
25 – 54 years	139	49.5	145	68.1	155	58.7
55 – 64 years	55	19.6	32	15.0	51	19.3
> 64 years	43	15.3	16	7.5	22	8.3
Male**	130	46.3	113	53.1	167	63.3
Race/Ethnicity***						
Black/African American	97	34.5	54	25.4	143	54.2
Latinx/Hispanic	119	42.3	121	56.8	65	24.6
White	44	15.7	29	13.6	39	14.8
Asian	18	6.4	7	3.3	14	5.3
Other	23	8.2	10	4.7	26	9.8
Education*						
Less than a high school degree	61	21.7	83	39.0	66	25.0
High school diploma or General Education Diploma	97	34.5	55	25.8	108	40.9
More than high school	122	43.4	73	34.3	86	32.6
Primary Language***						
English	197	70.1	100	46.9	221	83.7
Spanish	76	27.0	105	49.3	35	13.3
Other	8	2.8	7	3.3	7	2.7
English speaking proficiency***						
Well/very well	225	80.1	124	58.2	237	89.8
Not well/not at all	54	19.2	89	41.8	25	9.5
Veteran	8	2.8	7	3.3	11	4.2
Main Insurance***						
None	26	9.3	20	9.4	12	4.5
Medi-Cal	104	37.0	95	44.6	152	57.6
Medicare	56	19.9	19	8.9	39	14.8
Private	64	22.8	65	30.5	47	17.8
Other public insurance	31	11.0	14	6.6	14	5.3
Physical or mental disability affecting activities of daily living***	34	12.1	47	22.1	12	4.5
HIV positive	5	1.8	3	1.4	7	2.7

Statistically significant differences between housing status are indicated as follows:

* p< 0.05,

** P< 0.001,

*** P<0.0001.

*HIV*, human immunodeficiency virus.

**Table 3:** Prevalence of homelessness and housing instability.

	PRAPARE	AHC	Modified DHHS	Aggregate measure
Homeless	26%	24%	29%	35% (95% CI, 31–38)
Unstably Housed	25%	22%	13%	28% (95% CI, 25–31)

*PRAPARE*, Protocol for Responding to and Assessing Patients’ Assets, Risks, and Experiences; *AHC*, Accountable Health Communities; *DHHS*, Department of Health and Human Services; *CI*, confidence interval.
